# Rationale for Using Social Media to Collect Patient-Reported Outcomes in Patients with Celiac Disease

**DOI:** 10.4172/2161-069X.1000166

**Published:** 2014-02

**Authors:** KT Park, Merissa Harris, Nasim Khavari, Chaitan Khosla

**Affiliations:** 1 Division of Gastroenterology, Hepatology, and Nutrition, Department of Pediatrics, Stanford University School of Medicine, Stanford, CA, USA; 2 Center for Health Policy/Primary Care Outcomes Research, Stanford University, Stanford, CA, USA; 3 School of Medicine, George Washington University, District of Columbia, USA; 4 School of Medicine, George Washington University, District of Columbia, USA

**Keywords:** Social media, Social networking, Facebook, Patient-reported outcomes, Healthcare, Mobile technology, Quality-of-life

## Abstract

Patients with celiac disease (CD) are increasingly interconnected through social media, exchanging patient experiences and health-tracking information between individuals through various web-based platforms. Social media represents potentially unique communication interface between gastroenterologists and active social media users – especially young adults and adolescents with celiac disease-regarding adherence to the strict gluten-free diet, gastrointestinal symptoms, and meaningful discussion about disease management. Yet, various social media platforms may be underutilized for research purposes to collect patient-reported outcomes data. In this commentary, we summarize the scientific rationale and potential for future growth of social media in patient-reported outcomes research, focusing on college freshmen with celiac disease as a case study and provide overview of the methodological approach. Finally, we discuss how social media may impact patient care in the future through increasing mobile technology use.

## The Case for Social Media in Patient-Reported Outcomes Research

In this age of social media woven into the fabric of our technology-driven world, today’s physicians are re-examining established ideals of patient privacy and professionalism [1,2]. While the rise of social media within patient care raises real concerns, web-based applications – such as Facebook, Twitter, Google+, Doximity, web blogs, instant messaging platforms, and video chat – represent a growing medium for patients to interact with other patients and social media-savvy health care providers. In gastroenterology, the online celiac disease (CD) community is the largest and most established network of patients exchanging ideas and experiences [3,4]. Within reasonable organizational frameworks, the collective voice from the celiac community aims to raise awareness about important health policy issues affecting CD patients, such as dietary adherence to the gluten-free diet and the current dilemma of under diagnosing patients with silent CD [5,6].

Under the auspices of marketing strategy, researchers are gleaning abundant personal information about patients through social media – to no one’s surprise. Yet, physician-led research is slowly acclimating to this new approach to collecting patient-reported data, although some investigators are increasingly open to new ways of collecting data through social media [4,5]. Physicians and researchers in gastroenterology could benefit from a wealth of possibilities through conducting systematic research through online sources. Facebook, for instance, has more than 1 billion active users worldwide who spend nearly an hour online every day [6]. Evidence suggests that the majority of patients want their healthcare providers to incorporate social media in some patient care, including appointment scheduling and reminders, diagnostic test reporting, health information sharing, prescription notifications, and answering general questions [7,8]. In particular, since the average adolescent and young adult patient’s social media use is widely prevalent and willingness to engage providers through social media is generally favorable, our group hypothesized those biopsy-confirmed CD patients in this demographics may be an optimal group to perform a survey study using Facebook.

## Our Pilot Experience Collecting Patient Reported-Outcomes on Facebook

Based on visible interest from private biotechnology companies for clinical trials [9] and the National Institutes of Health [10], electronically-collected patient-reported outcomes (PROs) research is arguably one of the promising areas for future growth in clinical research. Given this background, our research group undertook an exploratory initiative using Facebook. Using our celiac center’s Facebook page (https://www.facebook.com/CeliacDiseaseLPCH), our research group successfully generated de-identified, longitudinal patient data from CD patients. We were granted institutional IRB approval for this prospective, cross-sectional pilot study. Over a 6 month study period, we collected approximately 150 separate survey responses from 32 college freshmen – 16 with CD and 16 age- and gender-matched healthy controls [9]. We aimed to assess changes in quality-of-life (QOL) and CD-related health due to difficulty adhering to a strict GFD during the first semester of college through 3 validated health surveys. At the end of our study, we saw no statistical difference in the QOL or overall health between the two groups. However, our notion was confirmed that de-identified databases for research were possible and reproducible by using Facebook as the primary portal for study participation.

Guiding principles of clinical research applies in-person and in social media. The planning phase is of utmost importance. We found 19 private or institutional celiac groups with an established presence on Facebook, as shown in Table 1 with each group’s total “Likes.” By contacting these groups through Facebook, information about our study was disseminated on many of the organizations’ Facebook page, and they often voluntarily generated separate web-based announcements, listserv emails, and other social media attention (e.g., Twitter) about our initiative. We adhered to a formal patient enrollment process and timeline, where posted electronic surveys on our Facebook page were used to determine study eligibility of potential participants [10]. HIPAA-compliant electronic surveys generated from an institutional survey tool (e.g., Qualtrics) are recommended. Whenever possible, as with any patient-oriented research, privacy and protection of patient-health information must take priority.

## Mobile Meets Value-Based Care and Patient-Reported Outcomes

There is a gradual but real momentum in clinical research to use PROs whenever possible. Health care is moving towards a value-based system, where improving patient-reported outcomes – such as QOL – are benchmarks of good clinical practice [11]. Therefore, clinical research naturally follows suite. Forward-thinking researchers can keep pace with this trend by realizing that the public has a natural demand for web-based modalities to facilitate patient care and research, especially on mobile devices. Celiac disease patients already utilizing daily social media use, especially among the adolescent and young adult patient population, represent an opportunity for clinician investigators in gastroenterology to gather valuable PROs.

Nearly 75% of all teens across all demographics use the internet from their mobile devices, and the majority of their total internet use is mobile [12]. There are approximately 7,000 (and growing) mobile healthcare applications in the Apple iTunes Healthcare Room. The future of social media seems to have a good foundation in mobile to effectively change the standard face-to-face and paper-trail methods of health care delivery. While increasing public demand will continue to upgrade the provider-patient experience and patient-advocacy interface of social media, effective methods of collecting PROs, on the other hand, may require more systematic collective thinking and future consensus.

## Figures and Tables

**Table 1:** Facebook Celiac Groups with “Likes” count as of April 9, 2013.

Organization	Web Location	#Facebook Likes (43,709 total)
National Celiac Foundation	http://www.facebook.com/NFCeliacAwareness	20,203 likes
Celiac Disease Foundation	http://www.facebook.com/CeliacDiseaseFoundation	13,742 likes
Univ of Chicago Celiac Disease Center	http://www.facebook.com/CureCeliac?ref=pb	12,740 likes
Gluten Intolerance Group of N. Am	http://www.facebook.com/GlutenIntoleranceGroup?ref=pb	8,404 likes
Center for Celiac Research	http://www.facebook.com/pages/Center-for-Celiac-Research/128798800407	3,518 likes
Celiac Sprue Association	http://www.facebook.com/csaceliacs	3,265 likes
Celiac Disease	http://www.facebook.com/pages/Celiac-Disease/129385497604?ref=pb	2,531 likes
American Celiac Disease Alliance	http://www.facebook.com/AmericanCeliacAlliance	1,792 likes
About Celiac Disease	http://www.facebook.com/pages/Aboutcom-Celiac-Disease/23213767439	1,494 likes
Gluten Free Bay	http://www.facebook.com/pages/Gluten-Free-Bay/22172143920?ref=pb	1,413 likes
Celiac Support Group at Boston Children’s Hospital	http://www.facebook.com/ChildrensCeliac?ref=pb	958 likes
g-free kids	http://www.facebook.com/gfreekid?ref=pb	656 likes
Gluten Free Kids	http://www.facebook.com/GFKblog?ref=pb	1,207 likes
The Gluten Free Student Cookbook	http://www.facebook.com/pages/The-Gluten-Free-Student-Cookbook/149325515133908?ref=pb	377 likes
Teens living with Celiac	http://www.facebook.com/TeensLivingwithCeliac?ref=pb	146 likes
Gluten Free College girl	http://www.facebook.com/pages/Gluten-Free-College-Girl/153799871335842	138 likes
Udi’s Gluten Free College Ambasasdors	http://www.facebook.com/pages/Udis-Gluten-Free-College-Ambassadors/178207262286166	92 likes
North Bay Celiac Group	http://www.facebook.com/NorthBayCeliacs	74 likes
Gluten Free College Kid	http://www.facebook.com/pages/The-Gluten-Free-College-Kid/229616083752941	54 likes

## References

[1] MacDonald J, Sohn S, Ellis P (2010). Privacy, professionalism and Facebook: a dilemma for young doctors. Med Educ.

[2] Guseh JS, Brendel RW, Brendel DH (2009). Medical professionalism in the age of online social networking. J Med Ethics.

[3] McNally Shawna L, Donohue Michael C, Newton Kimberly P, Ogletree Sandra P, Conner Kristen K (2012). Can Consumers Trust Web-Based Information About Celiac Disease? Accuracy, Comprehensiveness, Transparency, and Readability of Information on the Internet. Interact J Med Res.

[4] Hawn C (2009). Take two aspirin and tweet me in the morning: how Twitter, Facebook, and other social media are reshaping health care. Health Aff (Millwood).

[5] Radha SS, Caplan N, St Clair Gibson A, Shenouda M, Konan S (2012). Can patients really make an informed choice? An evaluation of the availability of online information about consultant surgeons in the United Kingdom. BMJ Open.

[6] (2013). Facebook Statistics.

[7] Fisher J, Clayton M (2012). Who gives a tweet: assessing patients’ interest in the use of social media for health care. Worldviews Evid Based Nurs.

[8] Chretien KC, Kind T (2013). Social media and clinical care: ethical, professional, and social implications. Circulation.

[9] Allison M (2012). Reinventing clinical trials. Nat Biotechnol.

[10] Gwaltney CJ, Shields AL, Shiffman S (2008). Equivalence of electronic and paper-and-pencil administration of patient-reported outcome measures: a meta-analytic review. Value Health.

[11] Hoffman A, Emanuel EJ (2013). Reengineering US health care. JAMA.

[12] Madden M, Lenhart A, Duggan M, Cortesi S, Gasser U (2013). Teens and Technology 2013. Pew Internet & American Life Project.

